# Unexpected Increase in Benzodiazepine Prescriptions Related to the Introduction of an Electronic Prescribing Tool: Evidence from Multicenter Hospital Data

**DOI:** 10.3390/diagnostics9040190

**Published:** 2019-11-15

**Authors:** Rosaria Del Giorno, Carmen Schneiders, Kevyn Stefanelli, Alessandro Ceschi, Sandor Gyoerik-Lora, Irene Aletto, Luca Gabutti

**Affiliations:** 1 Department of Internal Medicine, Regional Hospital of Bellinzona and Valli, 6500 Bellinzona, Switzerland; carmen.schneiders@gmx.ch (C.S.); Sandor.Gyoerik-Lora@eoc.ch (S.G.-L.); Irene.Aletto@eoc.ch (I.A.); 2 Department of Social Sciences and Economics, Sapienza University of Rome, 00186 Rome, Italy; kevyn.stefanelli@gmail.com; 3 Division of Clinical Pharmacology and Toxicology, Institute of Pharmacological Sciences of Southern Switzerland, Ente Ospedaliero Cantonale, 6900 Lugano, Switzerland; Alessandro.Ceschi@eoc.ch; 4 Department of Clinical Pharmacology and Toxicology, University Hospital Zurich, 8091 Zurich, Switzerland; 5 Institute of Biomedicine, University of Southern Switzerland, 6900 Lugano, Switzerland

**Keywords:** electronic prescribing tool, Benzodiazepines, prescriptions, hospital, increase, unexpected

## Abstract

Electronic Prescribing tools (e-prescribing) have shown several benefits in terms of prescribing process adequacy and health care quality in hospital settings. We hypothesize however, that an undesired effect of digitalisation, due to the easier and faster prescribing process allowing patients to skip face-to-face conversations with patients and nurses, is that it could facilitate the prescription of medications at high risk of overuse or abuse, such as benzodiazepines (BZDs). We conducted a panel data study to investigate, the impact of the introduction of an e-prescribing system on new BZD prescriptions in hospitalised patients in a network of five teaching hospitals. During the observation period 1 July 2014–30 April 2019, 43,320 admissions were analysed. A fixed-effects model was adopted to estimate the effect of e-prescribing on new BZD prescriptions. E-prescribing implementation was associated with a significant increase of new BZD prescriptions: absolute +1.5%, and relative +43% (*p* < 0.001). The effect was similar in males and females (respectively, absolute +2.3%, relative +65% (*p* < 0.001); absolute +1.8%, relative +58% (*p* = 0.01)) and in patients ≥70 years old (absolute +1.6%, relative +59% (*p* < 0.001)). After controlling for time-varying explanatory variables, the implementation of the e-prescribing tool showed similar significant effects. E-prescribing implementation was associated with a significant increase of new in-hospital BZD prescriptions. For classes of drugs at risk of overuse or abuse, e-prescribing should be used cautiously, to minimize the risk of over-prescriptions. Further research in other settings and countries is needed to analyse causal interactions between e-prescribing and BZD prescriptions in the hospital setting, and to promote the ultimate goal of high-value care.

## 1. Background

The implementation of electronic prescribing tools (e-prescribing) has become increasingly common in hospital settings worldwide. Previous studies have investigated the effect of introducing e-prescribing at the hospital level, exploring its impact on patient care quality by analysing outcome and safety parameters (guideline adherence, surveillance, and monitoring adequacy), and on prescription and dispensing errors [1,2,3,4,5]. Overall, the favourable effect of e-prescribing was due to improvement in prescription safety, mainly as a consequence of integrated alert systems that support physicians during the prescription process in case of drug interactions or inappropriate dosage (i.e., clinical decision support systems) [6,7]. Moreover, e-prescribing could be effective in promoting prescription stewardship, allowing real-time review and targeted interventions [8]. Nevertheless, controversial findings have also emerged, suggesting that the impact of e-prescribing is not always positive. Other studies have, in fact, surprisingly highlighted the onset of further medication-related prescription errors and an increase in mortality at the hospital level after the introduction of e-prescribing [9,10]. Furthermore, available previous reports on the cost-effectiveness of in-hospital e-prescribing are inconclusive—even more so considering that a limited number of studies on the topic have been conducted [11]. Last but not least, it is important to note that, paradoxically, e-prescribing tools in the out-of-hospital setting, through online pharmacies and electronic ordering platforms, have been associated with an increase of medication consumption by patients, mainly due to the abatement of physician-linked prescription barriers [12,13]. Considering the easier access to medication associated with e-prescribing in outpatient care settings, an important issue arises: could e-prescribing be associated with medication over-prescription in the hospital setting?

We hypothesized, in fact, that the digitalisation of prescribing in-hospital could lower the prescription threshold of certain classes of medications, mainly because ordering is simpler and faster and does not require being on the ward, offering a shortcut to skip conversations with nurses and patients, which is important for preventing potential overuse.

The risk of facilitating the prescribing process could be more evident for specific classes of drugs, such as benzodiazepines (BZDs), for which a public health problem of overuse has been raised and the risk of over-prescribing is significant and multifactorial (e.g., new prescriptions at the hospital level mostly performed during the night; prescription renewal as the simplest way to close the challenge of de-prescribing) [14]. Speaking of which, we could hypothesize that e-prescribing, allowing the automatic export of medication prescribed during the hospitalization into the discharge documents, could facilitate the transition from a short-term use of BZDs to an out-of-hospital prescription.

Furthermore, to our knowledge, the impact of digitalisation on BZD prescribing in the hospital setting has still not been explored.

In the present study, we aim to investigate, using a natural experiment based on panel data, the impact of the implementation of an electronic prescribing tool on BZD prescriptions in BZD-treatment-naïve patients, in a network of five teaching hospitals in Switzerland. One of the advantages in studying the process was that the e-prescribing system was implemented at different times in the hospitals of the network.

## 2. Methods

### 2.1. Setting, Study Population, and Design

This study was performed in the internal medicine wards of a network of five teaching hospitals in southern Switzerland belonging to the Ente Ospedaliero Cantonale (EOC). All data concerning new BZD prescriptions in patients admitted from 1 July 2014 to 30 April 2019 were analyzed. The hospitals of the network were labelled with the first five capital letters of the alphabet (A, B, C, D and E). The e-prescribing system was implemented in each hospital at different times (Hospital A: October 2016; Hospital B: August 2017; Hospital C: October 2015; Hospital D: October 2016; Hospital E: January 2017). The data analysed were collected on the basis of the standard hospital monitoring and did not contain patient information. Demographic data (age, gender) and information on diagnoses and case mix (an indicator of illness severity used to calculate hospitals’ reimbursements) were collected. The study was compliant with the “Strengthening the Reporting of Observational Studies in Epidemiology (STROBE) Statement” guidelines [15]. According to Swiss law, studies based solely on anonymous secondary data do not require approval from an ethics review board [16]. The study was reviewed by the Swiss Ethics Committee, which confirmed that, involving anonymous secondary data only, the study was exempt from institutional board approval.

### 2.2. Data Analysis

Descriptive statistics were used to evaluate characteristics of the hospitals during the study period, using the median and interquartile range (IQR) or frequencies (%), as appropriate. Preliminarily, an interrupted time-series analysis was used to estimate the changes in levels and trends of new BZD prescriptions (BZD initiated during the hospital stay) before and after the e-prescribing system implementation at each of the five hospitals. For the time series regressions, the *β*0 coefficient (i.e., the level of new BZD prescriptions at the beginning of the observation), the *β*1 coefficient (i.e., the baseline trend before e-prescribing implementation), the *β*2 coefficient (i.e., the change in new BZD prescription level during implementation), the *β*3 coefficient (i.e., the change in the slope of the trend of new BZD prescriptions after implementation), standard errors, and associated *p*-values were calculated.

The e-prescribing system was progressively introduced in the hospitals starting from October 2015. The time variation in the adoption of the system allowed for a natural experiment to assess the impact of e-prescribing on new BZD prescriptions.

To quantify the impact of e-prescribing on new BZD prescriptions, we used a fixed-effects (FE) panel design, exploiting the within-hospital variation induced by the digitisation of the prescription process. The design allows for the assessment of whether hospitals adopting e-prescribing experienced changes in the trend of new BZD prescriptions, by analysing within-hospital changes over time and comparing those changes to the new BZD prescription trends among hospitals that still had not passed to the e-prescribing system. To carry out this analysis, we estimated fixed-effects ordinary least squares regression models, where the new BZD prescription rate was the dependent variable. This model directly accounts for dynamic factors that cause new BZD prescriptions to vary from hospital to hospital, as well as for stable, unmeasured factors that differ between hospitals.

The statistical model (FE Model 1) used was:

*y_it_* = *α_i_* + *β_ep_x_it_* + *u_it_*

where *y_it_* is the percentage of new prescriptions for each month (*t*) and each hospital; *α_i_* is the unknown intercept for each hospital (n hospital-specific intercepts); *β_ep_* is the coefficient assigned to the new prescriptions’ percentage dummy variable; *x_it_* is the independent variable; and *u_it_* is the error term.

In addition, we also included, as a control variable, the monthly mean hospital case mix, with the aim of capturing inter-hospital influences on new BZD prescriptions that were not included in any explanatory variables. In this way, it was possible to verify whether the patient conditions were constant throughout the observation period, and exclude the hypothesis that changes in prescriptions were due to patient illness severity.

In order to explore the impact of case-mix heterogeneity, the following fixed-effects model (FE Model 2) for panel data was used:

*y_it_* = *α_i_* + *β_ep_x_1,it_* + *β_casemix_x _2,t+_u_it_*

with *β_casemix_*and *x_2t_* representing the coefficient assigned to the monthly average case mix and its relative value, respectively.

To evaluate the contributions of the different categories of age and gender on new BZD prescriptions, we explored the effect of e-prescribing implementation separately in subjects ≥70 and <70 years old, and in males and females.

We tested the random effect model as an alternative model, rejected on the Hausman test, the formal test exploring differences between the coefficients in the time-varying explanatory variables, in order to discriminate between fixed-effect and random effects models (rejected at *p* < 0.001) [17].

All regression analyses used robust standard errors. The level of statistical significance was set at *p* ≤ 0.05. All analyses were performed using R Statistical Software, version 3.2.0 (available online: www.r-project.org, accessed on 14 November 2019).

## 3. Results

### 3.1. Descriptive and Interrupted Time Series Analyses

Descriptive characteristics of the in-hospital study population, before and after the implementation of the e-prescribing tool, are represented in Table 1. During the pre- and post-e-prescribing periods, 20,197 and 20,023 admissions were analysed respectively.

Similar characteristics were found among hospitals comparing pre- and post-e-prescribing implementation, in particular with regard to the percentage of females (Hospital A: 50.6% vs. 51.2%, respectively; Hospital B: 56.7% vs. 56.9%, respectively; Hospital C: 47.7% vs. 47.5%, respectively; Hospital D: 48.2% vs. 49.4%, respectively; Hospital E: 50.7% vs. 50.0%, respectively) and of patients already treated with BZD at admission (Hospital A: 32.6% vs. 33.9%, respectively; Hospital B: 29.2% vs. 30.5%, respectively; Hospital C: 31.3% vs. 31.8%, respectively; Hospital D: 30.3% vs. 30.9%, respectively; and Hospital E: 29.4% vs. 28.6%, respectively).

The percentages of new BZD prescriptions were as follows: Hospital A, 3.8% vs. 3.4%; Hospital B, 5.7% vs. 7.2%; Hospital C, 5.6% vs. 5.3%; Hospital D, 3.6% vs. 2.9%; and Hospital E, 3.3% vs. 3.2%. New BZD prescriptions across network hospitals were preliminarily inspected considering the different times in which the e-prescribing system was locally implemented. Hospitals were comparable in terms of number of beds, healthcare providers, and characteristics (Supplementary Table S1).

**Figure 1:**
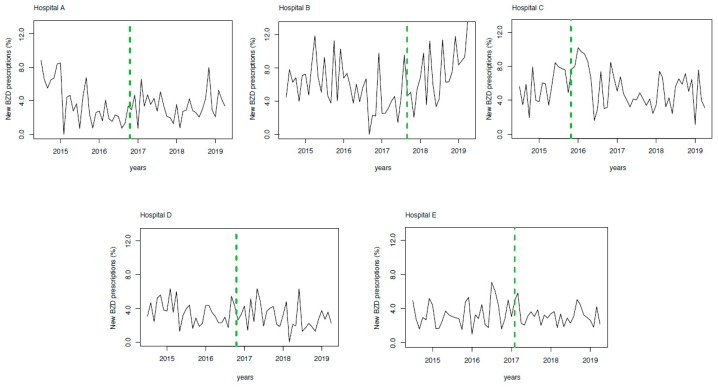
Benzodiazepine (BZD) prescriptions by hospitals before and after the e-prescribing tool implementation. Monthly rate of new BZD prescriptions during the period before and after the e-prescribing system implementation in hospitals A–E. Dashed green lines indicate the e-prescribing implementation start for each hospital.

The monthly rate of new BZD prescriptions by hospital is depicted in Figure 1, where the implementation time is indicated. Considering the three-month period immediately before versus after the intervention, the crude rate of new BZD prescriptions documented a percentage increase (Hospital A: 1.7% vs. 3.7%; Hospital B: 3.6% vs. 6.6%; Hospital C: 6.5% vs. 8.5%; Hospital D: 4.5% vs. 6.7%; and Hospital E: 4.4% vs. 3.6%).

An analysis of the interrupted time series confirmed a significant change in BZD prescriptions after implementation of the e-prescribing system in two of the hospitals (Hospital A: change in level *β* coefficient = 0.028, SE = 0.009, *p* < 0.001; trend change in *β* coefficient = 0.002, SE = 0.0005, *p* < 0.001; Hospital B: trend change in *β* coefficient = 0.004; SE = 0.001, *p* < 0.001) (Table 2).

### 3.2. Fixed-Effects Regression Models

To estimate the effect of e-prescribing implementation on new BZD prescription rates, two models were evaluated: the first without adjustments, and the second adjusted for case mix. The effect was estimated for the entire population and for subgroups based on age (</≥70 years old), and gender (male vs. female). Table 3 shows the coefficients based on the fixed effects (FEs) panel data regression models, signs, level of significance, and standard errors.

The results from the FE estimation approach shows that the coefficients of the effect of e-prescribing on new BZD prescriptions were positive for both models and in all subsets of patients, suggesting a causal relationship between e-prescribing and the increase in average inpatient BZD prescribing rates. Specifically, in all samples, the e-prescribing system was associated with a significant absolute and relative new BZD prescription increases of 1.5% and 43%, respectively (*p* < 0.001). The effect was even more pronounced in males, with absolute and relative increases of 2.3% and 65%, respectively (*p* < 0.001), as well as in subjects ≥70 years old, with values of 1.6% and 59%, respectively (*p* < 0.001). Considering the female subset separately, there was an increase of 1.8% and 58%, respectively (*p* = 0.01).

In the fixed effect model 2, adjusted by case mix, we found a similar pattern (see Table 3); even if in the entire sample population, the impact on new BZD prescriptions was not significant (absolute and relative values 1.4% and 37 %; *p* = 0.07). Nevertheless, the effect was clearly significant and similar in magnitude, to that it was found in the model without controlling, considering the subset of patients ≥70 years old (absolute increase = 2.8%; *p* < 0.001) and of the male gender (absolute increase = 2%, *p* = 0.03). In all fixed effects models, the variable implementation of e-prescribing showed a direct and significant relationship with new BZD presciptions.

**Figure 2:**
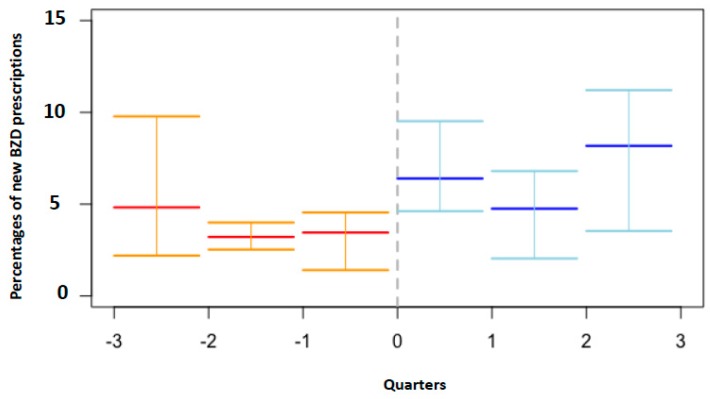
Benzodiazepine prescriptions before and after e-prescribing implementation at the network level. The zero on the *x*-axis indicates e-prescribing implementation. Quarterly rates of new BZD prescriptions in the period before and after e-prescribing are depicted in red and blue, respectively. The horizontal lines above and below the central value indicate the maximum and minimum BZD rates in the period. Percentages of new BZD prescriptions are depicted on the *y*-axis.

In Figure 1, the monthly rate of new BZD prescriptions by Hospitals A, B, C, D, and E are depicted, and the periods before and after the e-prescribing system implementation are marked. In Figure 2, the effect of e-prescribing implementation at the network level is represented. The figure clearly shows the increase of new BZD prescriptions after the implementation of the electronic tool.

## 4. Discussion

This study represents the first analysis of the effect of e-prescribing on specific classes of drugs at high risk of overuse/abuse, such as benzodiazepines, at the hospital level. Although previous reports have shown the efficacy of e-prescribing in improving the quality of care delivery [18], findings in this paper suggest a causal relationship between the implementation of the informatic tool in hospital wards and the simultaneous documented rise of BZD prescriptions. Specifically, our results demonstrate that e-prescribing increased inpatient BZD prescriptions by 42% in the period immediately following the implementation, with a higher impact in males and in subjects ≥70 years old.

In recent years, e-prescribing systems have emerged as tools, which together with other informatic medical technologies, such as electronic health records and networking, have been aimed at improving the quality and the personalisation of healthcare [19,20].

Overall, it is well known that, especially in outpatients, the benefits of e-prescribing are mainly related to patient safety, prescription adequacy, workflow efficiency, and cost savings [21].

As for outpatients, in the hospital setting, overuse and inappropriate prescribing of BZDs are critical issues, with previous reports indicating that BZD prescriptions in hospital are appropriate in only one-third of the cases [22,23].

Furthermore, inappropriate prescriptions in hospitals could impact patient outcome, leading to several consequences for patients (e.g., falls and fractures, delirium, paradoxical reactions, sleep disorders) [24,25]. The phenomenon of overuse or inappropriate prescribing of BZDs in hospitals could be related to several concurrent factors, but also to the tendency to renew previous prescriptions, creating a vicious circle [26].

Interventions of healthcare systems to curb new BZD prescriptions should be multifaceted, and should include a shared decision-making process based on a continuous conversation between patients and healthcare providers. In accordance with the principles of the “Choosing Wisely” campaign, which advocates high-value care for patients, we previously successfully tested an intervention aimed to curb new BZD prescriptions in hospitalised patients in our teaching hospital network [14].

With the present analysis, we brought to light a probably underestimated hidden issue, related to the possibility of increased prescribing of BZDs in hospitalised patients after the implementation of an e-prescribing tool. Our data support the existence of a causal relationship between the increase of BZD prescribing registered immediately after the e-prescribing implementation and its use in hospital wards. The impact was more pronounced in elderly patients (aged ≥70 years).

The use of a pseudo-experimental design was possible because the hospitals involved are part of a regional network. The network structure itself, in terms of clinical and administrative management characteristics, assures comparable hospital features (with similar workload and specialist resources), allowing us to consider each hospital as an internal control and to build panel data statistical models. Moreover, despite being located in separate hospitals, the wards involved in the study are part of the same internal medicine department. Internal shared guidelines include indications about the prescription of specific classes of drugs like BZDs. All the hospitals involved are teaching hospitals, with resident physicians following the same training track. The healthcare staff was similar between the hospitals, as well as patient characteristics.

Due to the cross-sectional nature of the investigation, we cannot elucidate the exact causes which explain the association between the introduction of the e-prescribing system and the related increase in BZD prescriptions. Nevertheless, we can speculate that the digitalisation of the prescribing process could directly affect the conversation between patients and healthcare providers for this class of medications. In fact, the old paper-based prescribing process previously used in the hospital wards could have, especially during the night-time shift, had a different impact on doctor–nurse and doctor–patient exchanges.

We hypothesize that the e-prescribing system implemented, based on the use of mobile devices like tablets, could facilitate the prescription of specific drugs like BZDs, especially in certain conditions (e.g., nighttime shifts, stressful working conditions, overwork), paradoxically reducing adequacy.

Similar to those obtained with other medication classes, an e-prescribing software including real-time targeted decision supports that alerts prescribers to the risk of prescription overuse could be useful in avoiding misuses of the prescription process [27,28,29]. Furthermore, previous studies have shown that digital alerts could be useful in reducing opioid and benzodiazepine co-prescribing among certain high-risk groups, suggesting that this system could also be useful in curbing the phenomenon of BZD overuse in the hospital setting [27,28,29].

As noted before, several reports have suggested that the implementation of e-prescribing tools could be part of the strategy to improve the quality of care [18]. Moreover, it is also known that sophisticated features integrated into some e-prescribing products (e.g., safety alerts, dose calculators, and medication selection aids) can assist healthcare providers in reducing error rates [30,31,32].

Coming back to the paper-based prescribing system previously in use, the risk of skipping the required face-to-face verification of the indication with the patient after the nurse call asking for a sleeping pill was further minimized by the fact that the prescription had to be filled at the unit level.

Here, we hypothesize that even if the contact with the patient is still required, the e-prescribing system offering a prescription shortcut could unmask, under special conditions related to workload and stress, the tendency to skip conversation. This aspect could become exacerbated during the night shifts, in which doctors who are not directly involved in the daytime activities are asked to prescribe reserve medications.

Furthermore, considering that the e-prescribing tool used, contrary to the previous prescribing process, is able to directly export the list of drugs prescribed during hospitalisation in the hospital discharge documents, the risk of an automatic continuation of the in-hospital medications could play a role.

We have to acknowledge some limitations in our study.

First, having been conducted in a network of teaching hospitals in southern Switzerland, findings should be interpreted in the investigated context, and may not be transferable to other medical facilities. Second, even if hospitals were comparable for patients’ characteristics, we cannot exclude a heterogeneity in physicians’ attitudes in BZD prescribing.

Moreover, we have to acknowledge that physicians in each hospital had a wide range of professional experience and different awareness of the potential BZD over/inappropriate use in the context of the ongoing “Choosing Wisely” campaign. Furthermore, some differences in BZD prescribing trends between hospitals emerged in the interrupted time series analysis. These differences in trends could be related to the fact that the principal investigator and promoter of the campaign has his main activity in one of the five hospitals in which the effect of e-prescribing was attenuated. Overall, the resulting trend in new BZD prescriptions after the e-prescribing introduction was probably partially mitigated due to the implementation, at the same time, of an intervention aimed at reducing new in-hospital BZD prescriptions [14].

We have to acknowledge that in the interrupted time series analysis, the change in level of new BZD prescriptions was significantly increased in only two hospitals, while in a third the registered increase was not statistically significant. Interrupted time series were indeed used as a preliminary analysis to explore the BZD prescription trend in each of the network hospitals. The significant associations found motivated the causality investigation, which was carried out using, in particular, fixed-effects (FE) panel analysis.

Regardless of the statistical point of view, some clinical and administrative aspects should also be mentioned. Although part of the same medical department, the hospitals are characterized by physicians with a wide range of professional experience and, as mentioned before, awareness of the potential BZD over/inappropriate use.

Furthermore, we have to acknowledge a further limitation of our study: evaluating the causal relationship between the introduction of e-prescribing and the registered increase in BZD prescription, due to the retrospective design, was not easy. This aspect has to be discussed, considering the ever-growing debate around the concept of evidence [33], which emphasizes the need for understanding the causal relationship in which evidence is obtained, and how the evidence could be interpreted and used in clinical decision making. We therefore believe that the impact of e-prescribing on BZD prescriptions in the hospital setting should be explored in other contexts and countries, with the aim of understanding the causal interaction and identifying correctable critical points.

With the present study, we are far from wanting to disown the advantages of e-prescribing in terms of traceability and safety. We feel, however, that considering the results of our study, integrating e-prescribing tools with alerts for drugs potentially at risk of abuse/misuse could be useful. Last but not least, even if we strongly believe in the importance of technology in supporting and improving the care delivered by providers, we would like to emphasize that the risk of shortcutting conversations and postponing clinical encounters should be taken into account.

Despite these limitations, and the inherent difficulties in evaluating the causal relationship between e-prescribing and an increase in BZD prescriptions, our results should be considered when defining implementation policies for drug prescription digitalisation at the hospital level.

## 5. Conclusions

Our analysis suggests that the implementation of electronic medication prescribing systems in the hospital setting could significantly increase the prescription of benzodiazepines in BZD-naïve patients. The negative impact of e-prescribing found here could have been the consequence of a simplification of the process of prescribing or, in the worst case, of impoverished conversation between patients, doctors, and nurses. Further research in other settings and countries is needed to analyse causal interactions between e-prescribing and BZD prescriptions in the hospital setting, and to promote the ultimate goal of high-value care.

## Figures and Tables

**Table 1:** Study sample characteristics (total admissions: 43220; years 2014–2019).

**Before e-Prescribing Implementation**					
	**Hospital A**	**Hospital B**	**Hospital C**	**Hospital D**	**Hospital E**
Admissions, *n*	3947	3206	2654	4632	5758
Age, median IQR	76 (62–84)	77 (67−85)	73 (59−82)	77 (65-84)	75 (61-83)
Age groups, *n* (%) (admissions)					
<70 years	1392 (35.3)	910 (28.4)	1105 (41.6)	1506 (32.5)	2180 (37.9)
≥70 years	2555 (64.7)	2296 (71.6)	1549 (58.4)	3126 (67.5)	3578 (62.1)
Gender, females (%)	50.6	56.7	47.7	48.2	50.7
Case-mix (median, Q1−Q3)	0.72 (0.53−0.93)	0.79 (0.59−1.00)	0.67 (0.50−0.92)	0.71 (0−52−0.93)	0.67 (0.48−0.92)
BZD at admission, *n* (%)	32.6	29.2	31.3	30.3	29.4
New BZD prescriptions, %	3.8	5.7	5.7	3.6	3.3
**After e-Prescribing Implementation**					
Admissions, *n*	4182	1937	7245	4733	4926
Age, median IQR	78 (67−85)	80 (69−86)	75 (62−83)	77 (66−84)	76 (63−84)
Age groups, (admissions)					
<70, y, *n* (%)	1235 (29.5)	489 (25.2)	2704 (37.3)	1461 (30.9)	1745 (35.4)
≥70, y, *n* (%)	2947 (70.5)	1448 (74.8)	4541 (62.7)	3272 (69.1)	3181 (64.6)
Gender, females (%)	51.2	56.9	47.54	49.4	50.0
Case-mix (median, Q1−Q3)	0.71 (0.52−0.96)	0.75 (0.54−1.04)	0.74 (0.51−1.01)	0.72 (0.51−0.99)	0.65 (0.48−0.90)
BZD at admission, (%)	33.9	30.5	31.8	30.9	28.6
New BZD prescriptions, (%)	3.4	7.3	5.3	2.9	3.2

IQR: interquartile range; y: year.

**Table 2:** Interrupted time-series regression analysis of new benzodiazepine prescriptions among hospitals in the network.

Hospital A	*β* Coefficient	Standard Error	*p*-Value
Baseline level (*β*0)	0.005	0.007	0.495
Baseline trend of BZD prescriptions before e-prescribing (*β*1)	−0.002	0.004	<0.001 *
Change in level at the implementation (*β*2)	0.028	0.009	<0.001 *
Trend change after the implementation (*β*3)	0.002	0.0005	<0.001 *
**Hospital B**			
Baseline level (*β*0)	0.047	0.005	<0.001 *
Baseline trend of BZD prescriptions before e-prescribing (*β*1)	−0.0008		0.026 *
Change in level at the implementation (*β*2)	−0.0389	0.0215	0.076
Trend change after the implementation (*β*3)	0.004	0.001	<0.001 *
**Hospital C**			
Baseline level (*β*0)	0.097	0.024	<0.001 *
Baseline trend of BZD prescriptions before e-prescribing (*β*1)	0.001	0.001	0.096
Change in level at the implementation (*β*2)	−0.038	0.025	0.017 *
Trend change after the implementation (*β*3)	−0.002	0.001	0.028 *
**Hospital D**			
Baseline level (*β*0)	0.029	0.005	<0.001 *
Baseline trend of BZD prescriptions before e-prescribing (*β*1)	−0.0004	0.0003	0.204
Change in level at the implementation (*β*2)	0.007	0.007	0.336
Trend change after the implementation (*β*3)	−0.000025	0.0004	0.958
**Hospital E**			
Baseline level (*β*0)	0.037	0.004	<0.001 *
Baseline trend of BZD prescriptions before e-prescribing (*β*1)	0.0002	0.0002	0.316
Change in level at the implementation (*β*2)	−0.0008	0.007	0.902
Trend change after the implementation (*β*3)	−0.0005	0.0004	0.206

BZD: benzodiazepine; * *p* < 0.05.

**Table 3:** Effect of e-prescribing implementation on new BZD prescriptions in the entire population by age classes and gender.

**Fixed Effect Model 1**										
	**Entire sample**		**<70 years**		**≥70 years**		**Males**		**Females**	
	Estimate (SE)	*p*-value	Estimate (SE)	*p*-value	Estimate (SE)	*p*-value	Estimate (SE)	*p*-value	Estimate (SE)	*p*-value
Effect of e-prescribing on new BZD prescriptions	0.015 (0.005)	<0.001 *	0.007 (0.010)	0.459	0.016 (0.006)	0.010 *	0.023 (0.007)	<0.001 *	0.018 (0.007)	0.010 *
Intercept	0.035		0.056		0.027		0.035		0.031	
*R* ^2^	0.0279		0.002		0.028		0.0405		0.0297	
**Fixed Effect Model 2**										
	**Entire sample**		**<70 years**		**≥70 years**		**Males**		**Females**	
	Estimate (SE)	*p*-value	Estimate (SE)	*p*-value	Estimate (SE)	*p*-value	Estimate (SE)	*p*-value	Estimate (SE)	*p*-value
Effect of e-prescribing on new BZD prescriptions	0.014 (0.008)	0.007	0.089 (0.014)	0.527	0.028 (0.007)	<0.001 *	0.020 (0.009)	0.035 *	0.004 (0.009)	0.891
Case mix × new BZD prescriptions	−0.004 (0.005)	0.363	−0.008 (0.006)	0.202	0.003 (0.004)	0.477	−0.003 (0.004)	0.544	−0.008 (0.004)	0.006
e-prescribing on New BZD prescriptions × case mix	0.006 (0.005)	0.251	0.006 (0.008)	0.448	−0.003 (0.004)		−0.002 (0.005)	0.978	0.008 (0.005)	0.063
Intercept	0.038		0.066		0.022		0.035		0.045	
*R* ^2^	0.068		0.021		0.082		0.036		0.031	

SE: standard error; * *p* < 0.05.

## Supplementary Materials

The following are available online at https://www.mdpi.com/2075-4418/9/4/190/s1.

## Abbreviations

EOC	Ente Ospedaliero Cantonale
BZDs	benzodiazepines
e-prescribing	electronic prescribing systems
FE	fixed effect
