# Supplementary material for: Unexpected Increase in Benzodiazepine Prescriptions Related to the Introduction of an Electronic Prescribing Tool: Evidence from Multicenter Hospital Data
Source: Diagnostics (Basel). 2019 Nov 15;9(4):190. doi: 10.3390/diagnostics9040190 (PMC6963612; doi:10.3390/diagnostics9040190)
Supplement: Supplementary file 1 [file diagnostics-09-00190-s001.pdf]

Suppl. Table 1. Number of beds, healthcare providers and characteristics by hospital (internal medicine only; total patient admitted, years 2014-2019, 43,220)

	**Hospital A**	**Hospital B**	**Hospital C**	**Hospital D**	**Hospital E**
Admissions, n	8129	5143	9899	9365	10684
Nurses, n	84	45	107	90	110
Physicians, residents, n	14	9	29	14	29
Senior phisicians, n	13	5	17	13	12
Number of hospital beds , n	73	49	77	60	73
